# Geo-resistivity data set for groundwater aquifer exploration in the basement complex terrain of Nigeria, West Africa

**DOI:** 10.1016/j.dib.2020.105975

**Published:** 2020-07-04

**Authors:** Wasiu Olanrewaju Raji, Kamil A. Abdulkadir

**Affiliations:** a Department of Geophysics, University of Ilorin PMB 1515, Ilorin, Nigeria; b Department of Geology and Mineral Sciences, University of Ilorin PMB 1515, Ilorin, Nigeria

**Keywords:** Groundwater, Aquifer potential, Vertical electrical sounding data, Electrical resistivity method, hydro-geophysics, Basement complex of Nigeria

## Abstract

To delineate prolific groundwater aquifers in the basement complex terrain of Ilorin, Nigeria, West Africa, a carefully designed Electrical Resistivity Surveys were undertaken at eighty-five (85) stations using Vertical Electrical Sounding (VES) technique with Schlumberger array. Data acquisition system comprised SAS 3000 ABEM Resistivity Meter, a D. C. battery, four metallic electrodes, electrical cables, portable Geographic Positioning System (GPS), hammers, measuring tapes, and other accessories. In addition to the resistivity data, the geographic coordinates of the survey points were measured for ease of geo-referencing of the exact points where the data were collected and for secondary use of the data. The data acquired were processed, analysed and interpreted using auxiliary curve matching techniques and computer iteration method. The results were used to: evaluate the subsurface in terms of the different geo-electric layers, their thicknesses, depths, and resistivities; evaluate the groundwater potential of the aquifer; identify suitable sites for locating boreholes for groundwater exploitation; and recommend depths to borehole bottom in the different parts of the study area. The data presented can be reused to determine the protective capacity of the overburden material against groundwater contamination; to determine the engineering-geophysics and geotechnical properties of the rocks for Civil Engineering applications; for predicting groundwater flow models; and for mineral exploration within the area. The data presented can be used with other geophysical and geological data acquired from the same area for a better evaluation of the subsurface for multipurpose applications.

Specifications Table

**Table:** 

Subject	Geophysics
Specific subject area	Electrical Resistivity Method
Type of data	Tables: vesdata.mat - MATLAB file containing raw VES data. : vesdata.cvs- CVS files containing raw VES data. : Appendix II - Excel file containing geo-reference data. Figures: (Appendix III(a) and Appendix III(b).
How data were acquired	Data were acquired using SAS 3000 ABEM Resistivity Meter, a D. C. battery, 4 metallic electrodes, electrical cables, GPS, and their assessories. Vertical Electrical Resistivity Sounding method of Schlumberger array was used to acquire the data. Data were acquired at eighty-five (85) locations covering the entire area of study.
Data format	Raw data Processed data images
Parameters for data collection	Data were collected at predetermined locations with inter-station spacing of 100 to 300 m where the metallic electrodes can be coupled to the ground for good contact and flow of electric current to the subsurface. Measurement points were selected to cover every parts of the study area. Current electrode separation, AB/2 ranges from 1 m to 100 m.
Description of data collection	Data collected are the electrical resistivities of soil and rocks in the subsurface of the area. The data is the reciprocal of conductivity which represent the opposition the rocks, at various depths, offer to the free flow of electrical current in the earth. The data can be inverted for the presence of water and natural resources in the subsurface.
Data source location	The data location is situated and lying between latitude 08° 28^I^ and 08° 33^I^ 15 ^II^ N of the equator and longitude 04° 27^I^ and 04° 32^I^ 40^.^E of the Greenwich Meridian. The area measures about 110 km^2^ in Ilorin, North central Nigeria, west Africa.
Data accessibility	The data set are with this article

## Value of the data

• The data are useful for characterizing the subsurface earth layer for groundwater exploration and exploitation, mineral exploration, and for civil engineering and geotechnical applications.
• Researchers and professionals in the field of groundwater, mineral exploration, construction and civil engineering can benefit from this data.
• The data can be used to determine the different geo-electric layers in the subsurface of the study area within the depth penetrated. Further, the data can be inverted to determine the depth and thickness of each geo-electric layer; to evaluate the groundwater potential of the aquifers, characterize fracture density in the subsurface, and predict depth to borehole for groundwater exploitation.
• This data set can be used for teaching and research purposes. It can also be used in the management of groundwater to prevent over-withdrawal that may cause aquifer damage, and in policy formulation.
• The data can be integrated with other geophysical and geological data for multipurpose applications in landfill siting, groundwater pollution control, dam site selection and other civil engineering applications [1], [2], [3], [4].
• The data can form a part of a large database or repository for regional hydrogeological study in Nigeria and Africa. Scarcity of data has been identified as the problem inhibiting comprehensive regional groundwater study in Africa [5], [6].

## Data description

The raw data are the electrical resistivities acquired by Vertical Electrical Sounding (VES) method. They are stored in file vesdata.mat and vesdata.cvs. The data represent changes in the resistivities of rocks and soil materials measured at various depths equivalent to AB/2. Where AB/2, the half of the current electrodes separation ranges from 1 to 100 m. The data acquisition equipment comprised SAS 3000 ABEM Resistivity Meter, four metallic electrodes, electrical cables, measuring tapes, portable Geographic Positioning System (GPS) device, hammer, battery, measuring tapes, a source of direct current (battery) and other accessories. The data is suitable for charactering subsurface rock layer/units and the features that are suitable for groundwater accumulation and mineral exploration. The first column of the MATLAB file contains the values of AB/2, while the rest 85 columns (2 to 86) contain the raw resistivity data acquired at the respective 85 locations. The same arrangement applies to the .CVS file. Appendix II is an excel file. It consists of geo-referencing data (the longitude and latitude), measured at every VES point using portable GPS device. The geo-referencing data are useful for locating the exact point where the electrical resistivity data were acquired. Geo-referencing data are important for field validation of the experiment and for identifying other geophysical data (e.g. Seismic or Ground Penetrating Radar) from the same area, that can be usefully combined in the future, for a better evaluation of the subsurface. Supplementary files III(a) and III(b) are Images data. Stored in .tiff format. These images are the plots of the processed data showing some groundwater aquifer parameters inverted from the raw data. Appendix III(a) is the thickness map of the weathered aquifers, while appendix III(b) is the estimated depths to the bottom of boreholes in the different parts of area. The image data provided useful insights to the hydrogeology of the study area. The data set is precise, repeated experiments at some locations within the area produced similar raw data sets and VES curves. The RMS error between the field data curve and the computer models curves is generally less than 10 percent.

## Experimental design, materials, and methods

### Study area

The study area is situated and lying between 08° 28^I^ and 08° 33^I^ 15 ^II^ N and longitudes 04° 27^I^ and 04° 32^I^ 40^.^E in Ilorin, north-central Nigeria, west Africa. The data area occupies about 110 km^2^ in Ilorin and falls within the Basement complex terrain of the northcentral Nigeria. The rocks in the area are considered to be Precambrian to lower Paleozoic in age [7], [8], [9]. The rock comprised mostly of gneiss, granite, schist, and undifferentiated meta-sediment. The oldest rock comprised gneiss complex whose principal member are biotite, hornblende, and gneiss [9] while the youngest suite are the younger granites having medium to coarse grains and variations in biotite content. In some places, the biotite minerals are thread-like, arranged in roughly parallel pattern, in other places, the biotite minerals are disoriented in the ground mass. The feldspar mineral occurs as fine-to-medium grains. Other rock types include schist and quartzite. Minor bodies of pegmatites and quartz intruded the main rocks at different places during the pan African Orogeny [10]. The rocks outcrop in a few places and are overlain by weathered rocks and top-soil in many places.

**Fig. 1:**
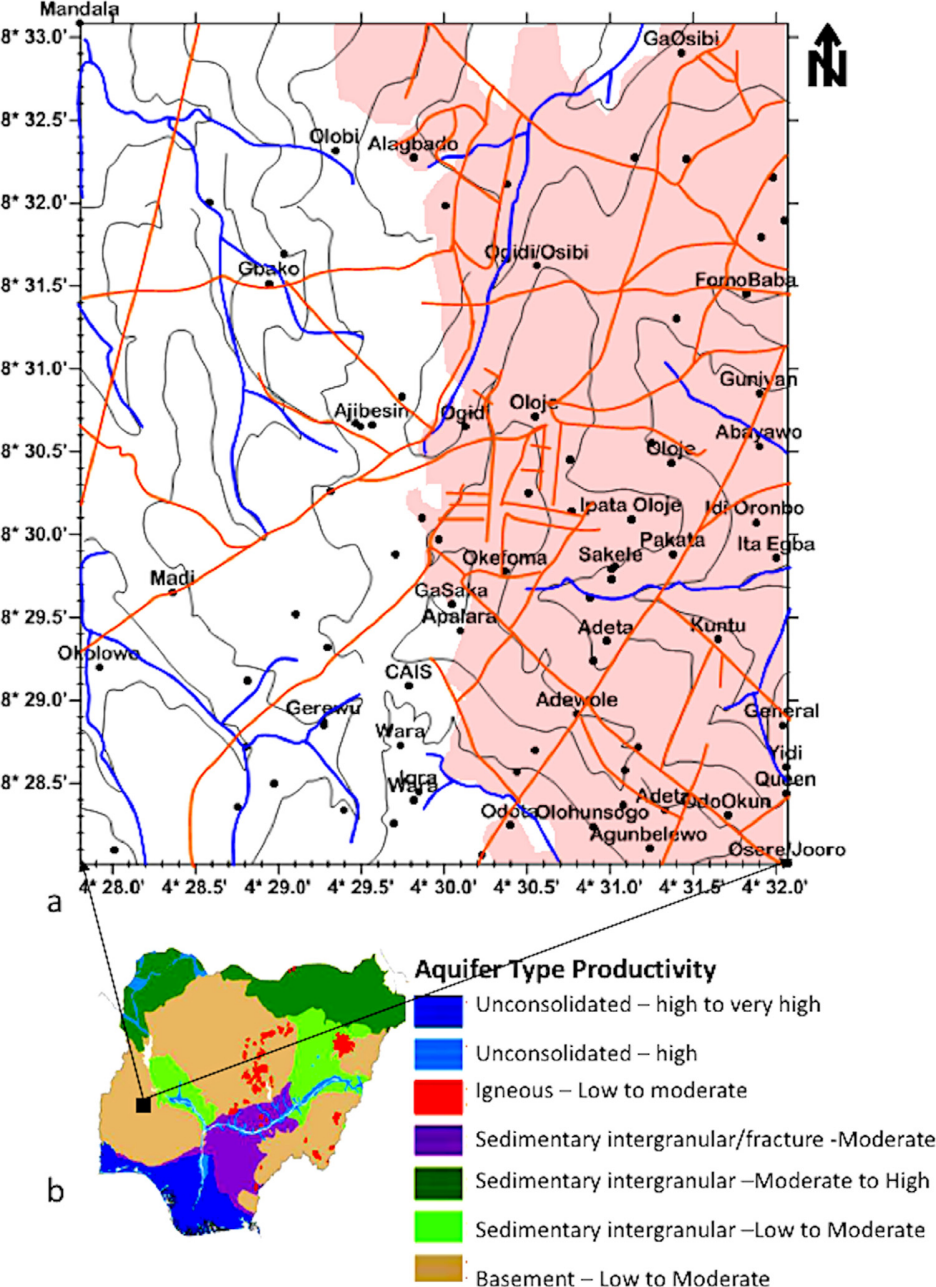
(a) groundwater aquifer productivity map of Nigeria [5], (b) Topographic map of the data area showing the locations of VES points.

Groundwater aquifers are formed by weathered rock, fractured rock, or a combination of weathered and fractured rocks [11], [12], [13] Fractured basement rock aquifers are found at higher depths compared to weathered rock aquifers. Although the weathered rock aquifers are more porous than the fractured rock aquifers, all basement rock aquifers have low porosity and permeability. They depend on secondary porosity caused by weathering, fracturing, jointing, cracking, and faulting to transmit fluids from one place to another. Geographically, the study area falls in the Savana Region of Nigeria. It has two major seasons: the dry and rainy seasons. Dry season usually falls within late October and early April, while rainy season falls within April and September. The mean annual temperature ranges between 27 and 35°, while the mean annual rainfall ranges from 75 to 112 mm. The main Drainage system in Ilorin is Asa River. Other smaller rivers in the area include Agba, Oyun, Aluko, Alalubosa, and Osere. The drainage pattern is dendritic and structurally controlled. Most of the rivers empty their water into Asa river which in turn empties its water into River Niger [14]. The topographic map of the study area is shown in Fig. 1.

### Data acquisition

Electrical resistivity data were acquired through a method known as vertical Electrical Sounding using the Schlumberger electrode array. Four metallic electrodes were coupled to the ground and arranged on a straight line. Current is introduced to the ground through a pair of electrodes separated by a distance AB, and the resulting potential is measured through another repair of electrodes separated by a distance MN. The four electrodes were arranged on a straight line with electrodes MN arranged in between electrodes AB. The separation between MN is usually small compare to AB. MN is usually less than one-third of AB.

To take measurements, the electrodes are connected to the resistivity meter and then to a source of direct current (battery) using electrical cables.

To probe higher depths, the distance between the electrodes AB and MN (more frequently, AB) are increased. For every change in AB, resistance (*r*) is measured. In this experiment, AB was expanded gradually from 1 to 200 m. Resistivity (*ρ*) is obtained when the measured resistance is multiplied by the geometric factor (*G*). Where *G* is the separation between the electrodes. The resistivity is then recorded against the corresponding AB/2 as shown in the data in appendix I. Detailed information on the VES method of Electrical resistivity measurement and the field procedure can be found in some geophysics literature including [15], [16]. The experiment was designed such that measurements are taken at pre-selected points. VES locations were separated with distance ranging from 100 to 300 m depending on space availability. The VES points were distributed to cover every part of the study area to allow a thorough and inclusive evaluation of the subsurface. Data acquisition took place in March 2018 at the peak of dry season. The maps showing the study area and the VES location are shown in Fig. 1. The coordinates of the VES points are shown in Table 1.

**Table 1:** coordinates of the VES data points (longitude 8O and Latitude 4O.

VES no Easting Northing	VES1 28.37 29.65	VES2 3138 28.34	VES3 31.37 30.43	VES4 31.66 27.37	VES5 31.34 32.91	VES6 31.90 30.53	VES7 30.13 30.65	VES8 30.88 29.62	VES9 30.77 30.14	VES10 29.57 30.66	VES11 29.82 28.40
VES no Easting Northing	VES12 30.55 30.71	VES13 31.25 30.55	VES14 30.05 29.58	VES15 30.77 30.14	VES16 31.01 29.79	VES17 27.92 29.20	VES18 28.58 32.00	VES19 30.51 30.25	VES20 31.82 31.45	VES21 28.95 31.52	VES22 30.76 30.45
VES no Easting Northing	VES23 31.03 29.81	VES24 31.15 32.27	VES25 30.01 31.99	VES26 31.88 30.07	VES27 31.38 29.88	VES28 30.98 29.35	VES29 31.90 30.85	VES30 31.13 30.09	VES31 32.01 29.86	VES32 29.71 29.88	VES33 29.50 30.65
VES no Easting Northing	VES34 29.85 28.45	VES35 29.28 28.87	VES36 29.47 30.67	VES37 31.01 29.73	VES38 29.34 32.32	VES39 31.01 29.70	VES40 30.37 29.78	VES41 31.40 31.30	VES42 29.03 31.70	VES43 27.81 33.08	VES44 27.87 30.01
VES no Easting Northing	VES45 28.97 28.49	VES46 29.97 29.95	VES47 29.31 30.26	VES48 29.75 30.83	VES49 30.56 31.62	VES50 29.82 32.27	VES51 31.98 32.15	VES52 32.05 31.89	VES53 30.38 32.11	VES54 31.92 31.79	VES55 31.46 32.26
VES no Easting Northing	VES56 31.71 28.30	VES57 32.07 28.01	VES58 32.04 28.85	VES59 32.06 28.44	VES60 32.06 28.60	VES61 30.80 28.92	VES62 30.44 28.56	VES63 30.90 29.24	VES64 31.09 28.53	VES65 31.17 28.72	VES66 30.55 28.70
VES no Easting Northing	VES67 30.10 29.42	VES68 31.24 28.11	VES69 29.79 29.09	VES70 29.29 29.32	VES71 28.81 28.12	VES72 29.70 28.25	VES73 29.10 29.52	VES74 28.01 28.10	VES75 28.81 28.72	VES76 29.40 28.35	VES77 29.27 28.85
VES no Easting Northing	VES78 28.75 28.36	VES79 30.40 28.24	VES80 30.23 28.07	VES81 30.90 28.24	VES82 31.08 28.37	VES83 29.74 28.73	VES84 32.24 28.68	VES85 30.41 29.42			

## Data processing

The data collected were pre-processed (where necessary) to remove errors in the measurements due to instrument failure, low battery, or poor contact between electrodes and the ground. The data were interpreted using the auxiliary curve matching method. Resistivity data were plotted against AB/2 on double logarithm paper to produce a curve for each VES point on the field. The curves were transferred to transparent papers and matched with the theoretical curves [17], [18] to delineate the different geo-electric layers in the subsurface and their resistivities, thickness and depth. Results from the curve matching method and the field data were input into the computer iteration software – IPI2win software [19] for the final interpretation.

**Fig. 2:**
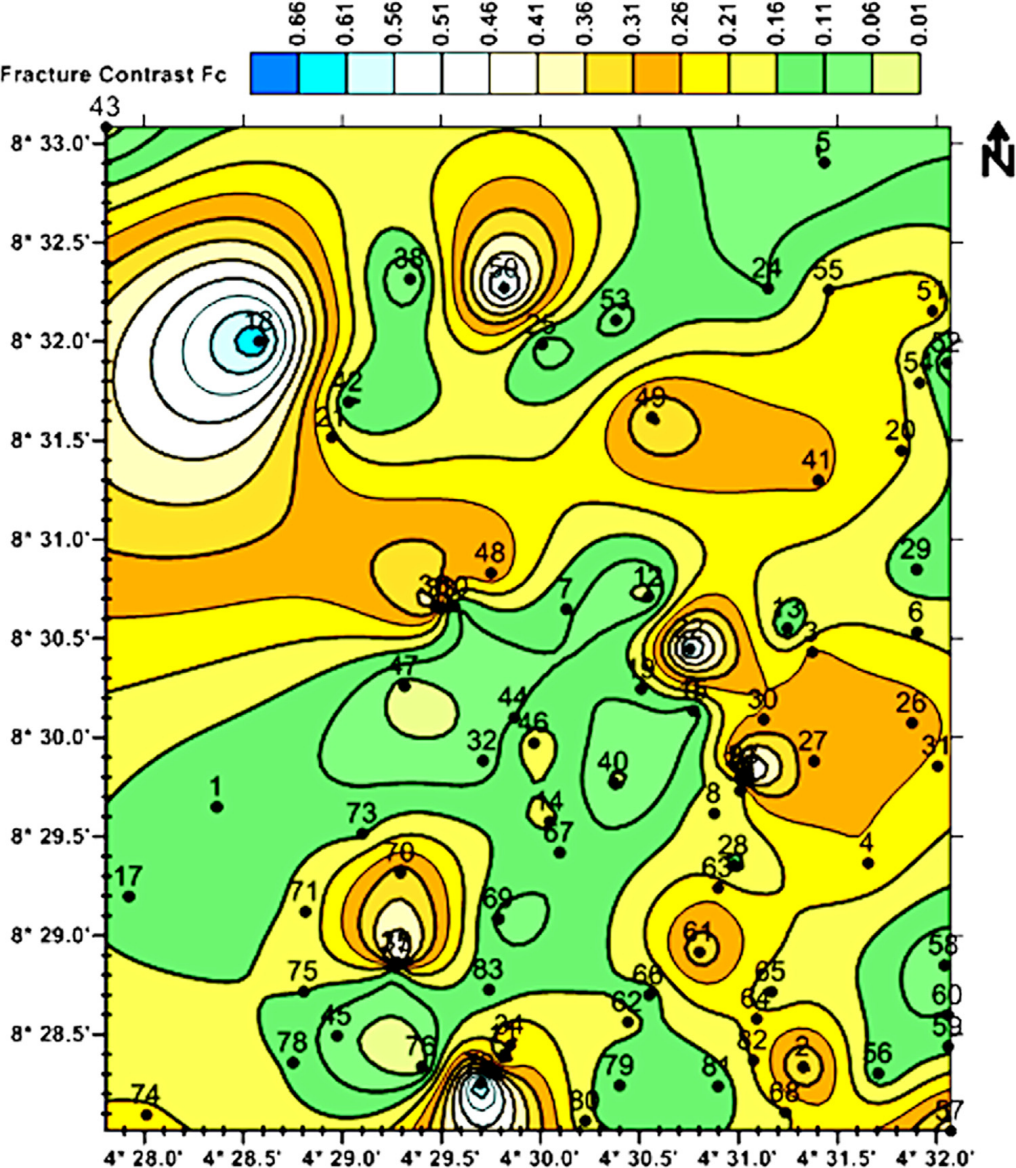
Fracture contrast of the weathered aquifers in the study area.

After a pre-set number of iterations, the software matches the curve from field data to a computer defined curve (an Earth model), and it outputs the estimated number of layers, the resistivities, thickness, and depth of each layer, and the RMS error. The RMS error defines the misfit or mismatch between the field data curve and the computer defined curve. Where the misfit was higher than 10%, the preset parameters, for examples, the number of layers or the number of iteration was reset and the interpretation process was repeated until the RMS error falls within a 1% and 10%. The final results – resistivity, thickness, and depth of each layer is usually plotted against the geographic coordinate of the VES points using contour maps to describe the spatial distribution of the layers and the aquifer properties [20], [21]. A spatial distribution map showing the fracture contrast in the weathered aquifer is presented in Fig. 2. This map is essential in the selection of points or locations for drilling boreholes. Fracture density is an essential parameter for identifying productive groundwater aquifers in the basement rock terrain. Other immediate applications of the data include determining the protection capacity of the overburden materials against groundwater pollution, determining the thicknesses of the aquifers, predicting depth to the bottom of boreholes in the different parts of the data area.

## Declaration of Competing Interest

The authors declared that there are no known competing financial interests, proprietary, or personal relationships that have, or could be perceived to have, influenced the work reported in this article.

## Appendix Supplementary materials

Image, image 1

Image, image 2
