# Supplementary figures and images for: Geo-resistivity data set for groundwater aquifer exploration in the basement complex terrain of Nigeria, West Africa
Source: Data Brief. 2020 Jul 4;31:105975. doi: 10.1016/j.dib.2020.105975 (PMC7358263; doi:10.1016/j.dib.2020.105975)

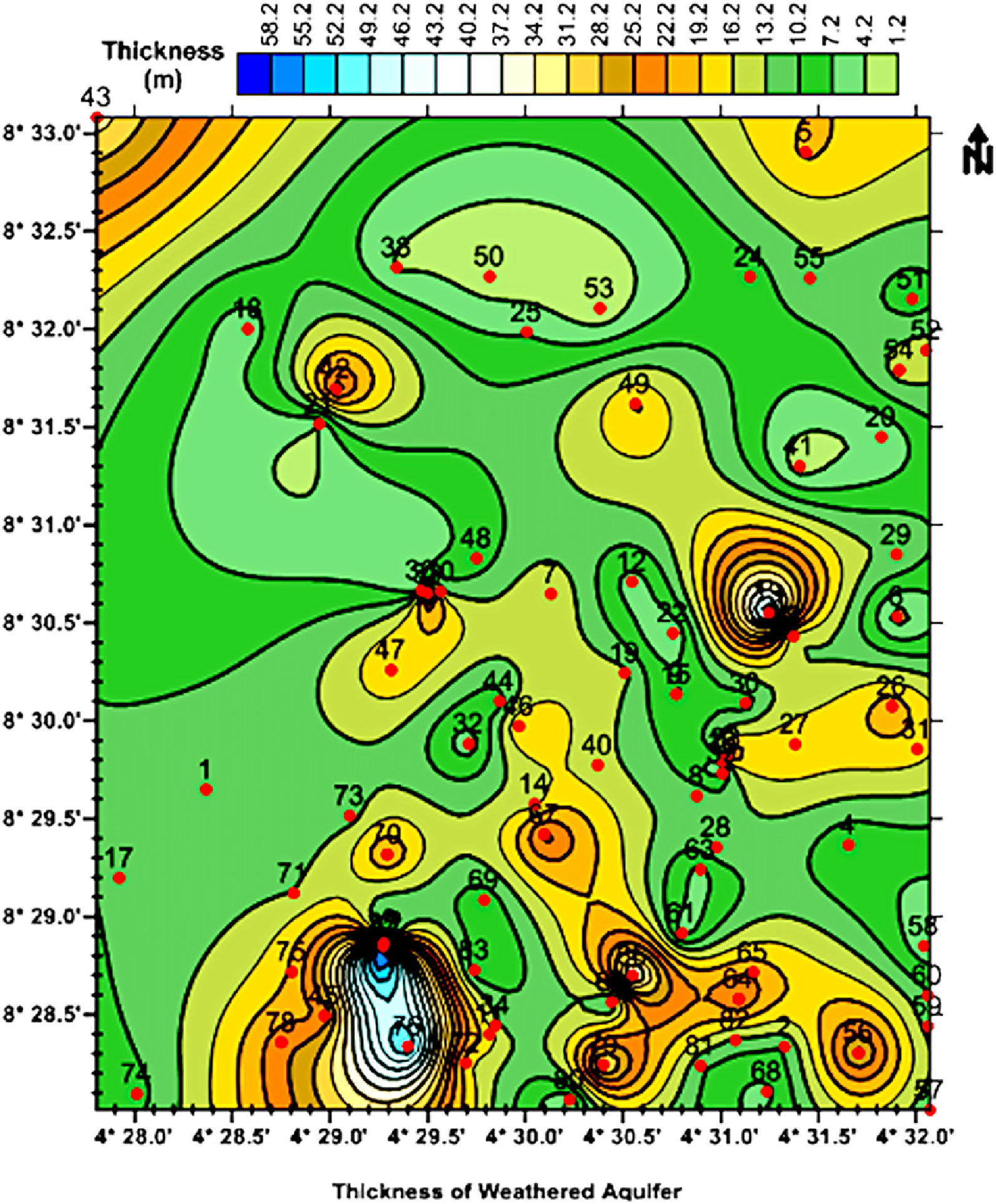

Supplement: Supplementary file 1 [file mmc1.jpg]

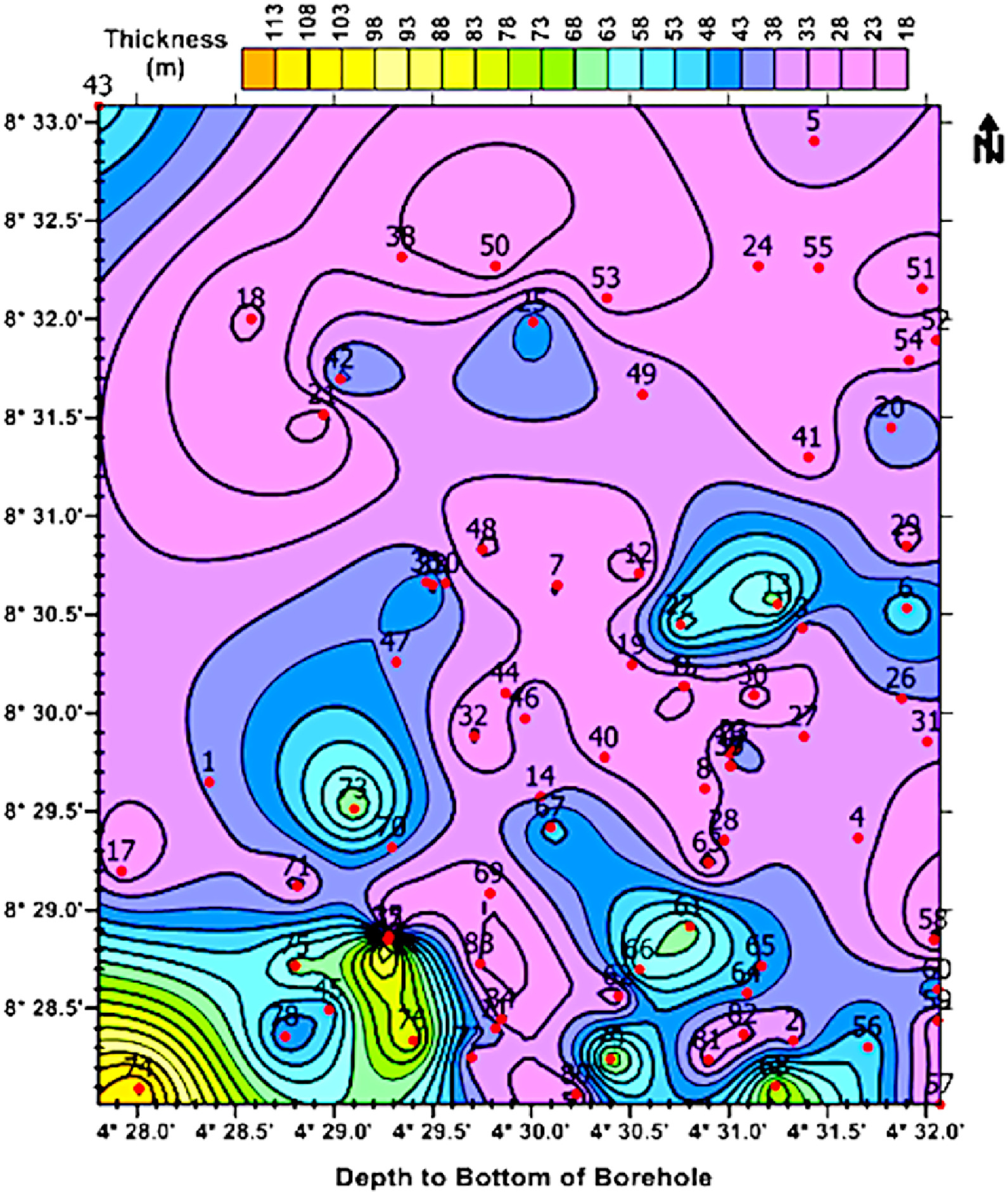

Supplement: Supplementary file 2 [file mmc2.jpg]
